# All-Fiber Tunable Pulsed 1.7 μm Fiber Lasers Based on Stimulated Raman Scattering of Hydrogen Molecules in Hollow-Core Fibers

**DOI:** 10.3390/molecules26154561

**Published:** 2021-07-28

**Authors:** Wenxi Pei, Hao Li, Wei Huang, Meng Wang, Zefeng Wang

**Affiliations:** 1 College of Advanced Interdisciplinary Studies, National University of Defense Technology, Changsha 410073, China; peiwenxi@nudt.edu.cn (W.P.); lihao18c@nudt.edu.cn (H.L.); cuiyulong@nudt.edu.cn (W.H.); wangmeng@nudt.edu.cn (M.W.); 2 Hunan Provincial Key Laboratory of High Energy Laser Technology, Changsha 410073, China; 3 State Key Laboratory of Pulsed Power Laser Technology, Changsha 410073, China

**Keywords:** hydrogen molecules, hollow-core fibers, fiber laser, stimulated Raman scattering

## Abstract

Fiber lasers that operate at 1.7 μm have important applications in many fields, such as biological imaging, medical treatment, etc. Fiber gas Raman lasers (FGRLs) based on gas stimulated Raman scattering (SRS) in hollow-core photonic crystal fibers (HC-PCFs) provide an elegant way to realize efficient 1.7 μm fiber laser output. Here, we report the first all-fiber structure tunable pulsed 1.7 μm FGRLs by fusion splicing a hydrogen-filled HC-PCF with solid-core fibers. Pumping with a homemade tunable pulsed 1.5 μm fiber amplifier, efficient 1693~1705 nm Stokes waves are obtained by hydrogen molecules via SRS. The maximum average output Stokes power is 1.63 W with an inside optical–optical conversion efficiency of 58%. This work improves the compactness and stability of 1.7 μm FGRLs, which is of great significance to their applications.

## 1. Introduction

The 1.7 μm band has unique spectral characteristics. This band is located in the valley between the absorption peaks of water molecules and is also located near the absorption peak of fat. As there are many water molecules and fats in biological tissues, 1.7 μm fiber lasers are widely used in the field of bio-imaging and medical treatment [1,2]. Moreover, the 1.7 μm band covers the absorption peaks of covalent bonds such as C-H bonds, so 1.7 μm fiber lasers have important applications in organic materials processing and gas detection [3,4]. Normally, there are mainly two kinds of methods to generate 1.7 μm band laser emission. One is to pump rare-earth-doped fibers, including thulium-doped fibers (TDFs) [5,6], bismuth-doped fibers (BDFs) [7,8], and thulium–holmium co-doped fibers (THDFs) [9,10]. However, the conversion efficiency of TDFs is relatively low due to strong reabsorption in the 1.7 μm band, while the preparation technology of BDFs and THDFs are not mature. The other is through nonlinear optical effects in solid-core fibers, including SRS [11,12], self-phase modulation [13,14], soliton self-frequency shift [15,16], and four-wave mixing [17,18], but the output laser linewidth is often wide. For some applications such as gas detection, narrow linewidth laser beams are needed to accurately distinguish the gas absorption lines, and a longer coherence length is also conducive to long-distance detection. The FGRLs generate laser beams based on gas SRS in hollow-core fibers (HCFs) [19,20,21,22,23,24,25,26], having the advantages of flexible wavelength tuning, narrow linewidth, and high conversion efficiency [27], which provide a new way to realize 1.7 μm band fiber laser output. In 2020, we first reported a 1.7 μm FGRLs based on hydrogen-filled HC-PCFs, but the maximum output power was less than 1 W [28]. Subsequently, based on an effective and reliable numerical model [29], we further improved the output power to 3.3 W by optimizing the experimental parameters, which is the highest average power of a 1.7 μm fiber lasers with a nanosecond pulse width [30]. However, in all reported 1.7 μm FGRLs, the output end of the HC-PCF is sealed in a bulky gas cavity, which is not conducive to their practical application. Therefore, it is necessary to realize the all-fiber structure FGRL to improve the stability and compactness of the system.

In this paper, we have demonstrated a tunable pulsed 1.7 μm FGRL with all-fiber structure for the first time. The 9-m-long gas cavity filled with 16 bar hydrogen is fabricated by fusion splicing a HC-PCF directly with solid-core fibers. When pumped by a homemade pulsed Erbium-doped fiber amplifier (EDFA) at 1.5 μm, efficient 1.7 μm laser emission is obtained based on the pure rotational SRS of hydrogen molecules. By adjusting the repetition frequency of the pump pulse, the maximum output Stokes power of 1.63 W at 1.7 μm is obtained with the repetition frequency of 1 MHz. The corresponding optical–optical conversion efficiency inside the gas cavity is 58%. This work improves the stability and compactness of the FGRLs, which is of great significance for its practical applications. 

## 2. Experimental Setup

**Figure 1:**
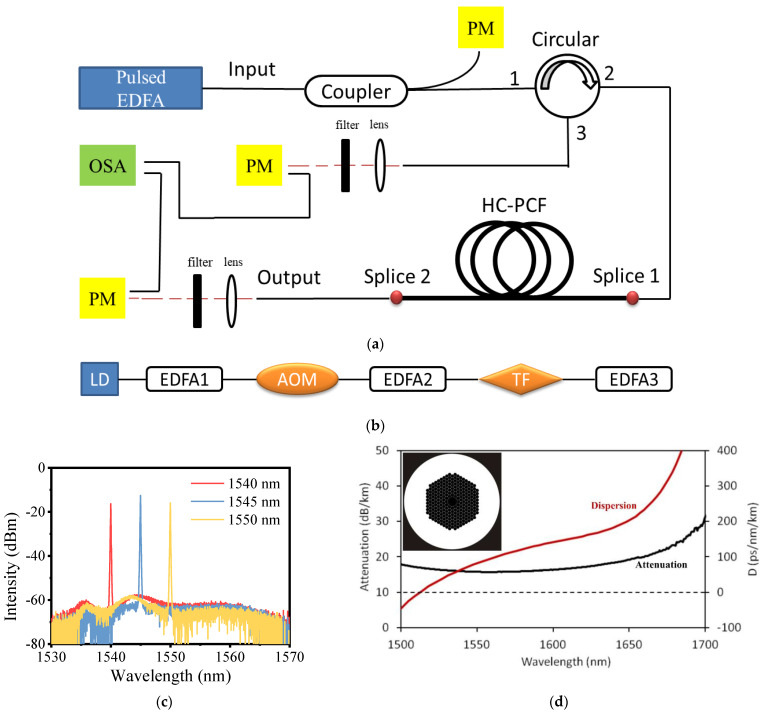
(**a**) Experimental setup: EDFA: Erbium doped fiber amplifier, PM: power meter, OSA: optical spectrum analyzer. (**b**) The structure of the pump source. TF: tunable filter. (**c**) The output spectrum of the pump source at maximum power. (**d**) The attenuation and dispersion curves of the HC-PCF, the insert: the optical microscope image of its cross section.

The experimental setup is shown in Figure 1a. The pump source is a homemade EDFA, which is the same as the one used in our previous work [30]. It consists of a laser diode, an acoustic optical modulator (AOM), a tunable filter, and three stages of amplifiers, as shown in Figure 1b. The seed is a laser diode (CobriteDX1, ID Photonics) and the continue wave (CW) laser generated by it can be amplified by EDFA1. Then, the CW laser is modulated into a pulsed laser by the AOM and is amplified through EDFA2. A tunable filter is used to filter out the possible amplifier spontaneous emission (ASE), and the EDFA3 amplifies the output power again. In this way, the homemade pulsed EDFA is tunable in the range of 1540~1550 nm with the maximum output power of ~7.5 W. We measured the power stability within 4000 s when the pump source operates with maximum output power, and the fluctuation ratio (the ratio of the difference between the maximum and minimum power to the average power in 4000 s) is less than 0.67%, which shows its good power stability. The repetition frequency ranges from 0.5 MHz to 10 MHz, and the pulse width is set to 10 ns. Figure 1c shows the output spectrum of the pump source with the maximum power. It can be seen that the ASE is well suppressed.

The pigtail of the pump source is fused with a fiber coupler (the measured coupling ratio is about 92.2: 7.8) which can monitor the real-time output power of the pump source. The main output end of the coupler is fused with an optical circulator so that the backward light can be measured. Figure 1d shows the attenuation and dispersion curves of the used HC-PCF (HC-1550-02, NKT Photonics) from the product specification, which shows a low-loss transmission range of 1500 nm to 1650 nm. The core diameter of the HC-PCF is ~10 μm, and the optical microscope image of its cross section is shown in the insert of Figure 1d. An all-fiber gas cavity is formed by two sections of solid-core single-mode fibers (SM28e, Corning) and the HC-PCF. One end of the HC-PCF is fused with a solid-core fiber directly by an arc discharge fusion splicer and this solid-core fiber is spliced to the circulator port2, which works as the injected end of the pump laser. The HC-PCF can be vacuumed through the other end by a vacuum pump and filled with high-pressure hydrogen. Then, it is fused with another solid-core fiber, which is the output end of the gas cavity. By calculating the time and gas leakage [31], the final pressure in the cavity can be estimated to be about 16 bar. The hydrogen in the all-fiber gas cavity is supposed to be kept at a high pressure so that the Raman gain can be saturated and provide high Raman gain. In our previous work [29], we demonstrated that high pressure above 10 bar can provide the saturated Raman gain, therefore 16 bar gas pressure is quite suitable. Besides, the splice loss at both ends of the cavity is 1.4 dB and 1.9 dB, respectively, as marked in Figure 1a (Splice1 and Splice2). Two convex-plane lenses are placed at the output end of the cavity and port3 of the circulator, respectively so that the output beam can be collimated. The filters after them have more than 95% transmission when the wavelength is above 1600 nm, meaning the pump laser and the Stokes laser can be separated easily. 

## 3. Experimental Results and Discussion

### 3.1. Spectrum Characteristics

**Figure 2:**
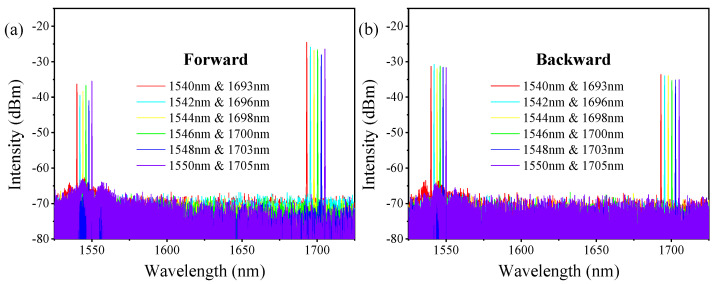
The output spectrum of (**a**) the forward and (**b**) the backward laser at the maximum pump power.

When the pump source is at the maximum output power and the repetition frequency is 1 MHz, the output spectrums of the forward and backward lasers are measured, as shown in Figure 2a,b, respectively. The pump lines range from 1540 nm to 1550 nm, and the first-order Raman lines range from 1693 nm to 1705 nm. Each pump line corresponds to only one Raman line with the Raman frequency shift coefficient of 587 cm^−1^. Due to the narrow low-loss transmission band of the HC-PCF, high-order Raman lines are strongly suppressed due to higher attenuation. Besides, Raman lines with a frequency shift coefficient of 587 cm^−1^ have a higher gain than the lines with other frequency shifts (like lines at 1640 nm with the shift coefficient of 354 cm^−1^) [32], thus pump lines are converted into these Raman lines preferentially. The backward pump lines and Raman lines have similar spectra to the forward lasers. These pump lines are mainly from the Fresnel reflection of the pump laser at splice1, while these Raman lines are reflected by splice2, as marked in Figure 1a. 

### 3.2. Measured Pulse Shapes

**Figure 3:**
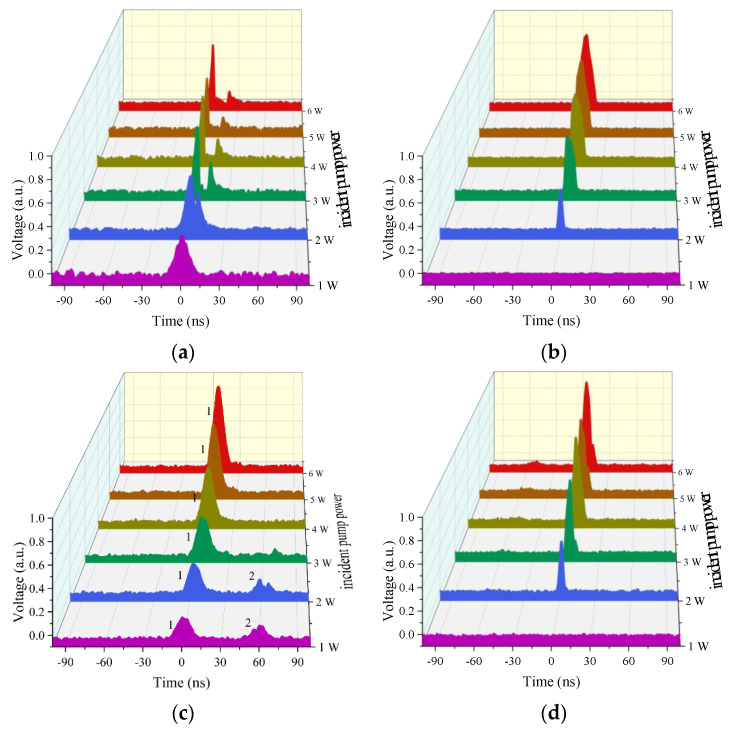
The output pulse shapes of (**a**) the forward pump laser, (**b**) the forward Raman laser, (**c**) the backward pump laser, and (**d**) the backward Raman laser.

By introducing two filters with different transmittance, the output pulse shapes of Raman laser and pump laser are measured, as shown in Figure 3. Here, we define the incident pump power as the output power of the circulator port2. Due to the insert loss of the circulator, the maximum incident power is limited to within 6 W. Figure 3a,b present the pulse shape evolutions of the forward pump laser and the Raman laser with the incident pump power. Obviously, with the increase in the incident pump power, the dip in the profile of the pump pulse becomes deeper and wider gradually. The reason is that higher incident pump power means higher peak power of the pump pulse and more energy beyond the Raman threshold is converted into Raman lasers. Therefore, as the incident pump power rises, the forward Raman pulse has a stronger intensity and a wider width, as shown in Figure 3b. Figure 3c,d present the shapes of the backward pump pulse and Raman pulse. It can be seen that both of them get stronger when the incident pump power rises up. Moreover, when the incident pump power is 1 W, there are two pump pulses detected with a time interval of ~60 ns, as marked by pulse1 and pulse2 in Figure 3c. They are pump pulses reflected by splice1 and splice2, respectively. The time interval accurately corresponds to the time of the pump pulse travelling forth and back in the 9-m-long HC-PCFs. We can see that pulse2 is much weaker than pulse1 as it transfers much longer distance and become weaker due to the transmission loss in the gas cavity. Pulse1 becomes stronger when the incident pump power increases as it is reflected by splice1 directly, while pulse2 becomes weaker. The reason is the increase in the Raman conversion, which causes less residual pump laser to be present in the gas cavity, which means less pump laser is reflected by splice2.

**Figure 4:**
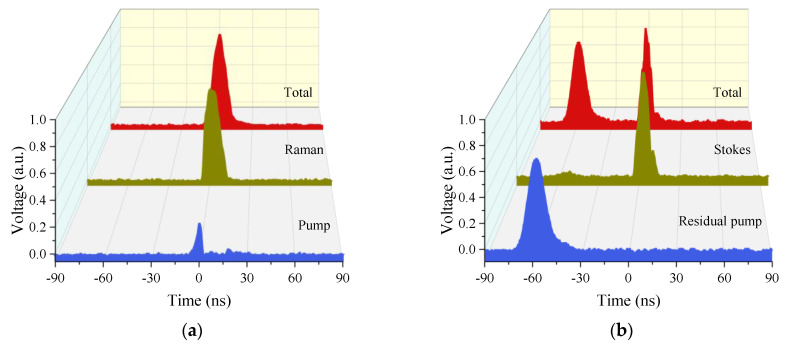
The pulse shapes of (**a**) the forward and (**b**) backward lasers with an incident power of 6 W and a repetition frequency of 1 MHz.

Moreover, we measured the pulse shapes without filters (named as total pulse shape) of forward and backward laser when the incident pump power is 6 W and the frequency is 1 MHz, as presented in Figure 4a,b. Both of them show similar characteristics to those analyzed above. However, the backward pulse cannot be detected at the same time, and the Raman pulse has a time delay of ~60 ns, as shown in Figure 4b. Thus, the Raman pulse is reflected by splice2 and the pump pulse is reflected by splice1 directly, which resembles the previous analysis.

### 3.3. Output Laser Power

**Figure 5:**
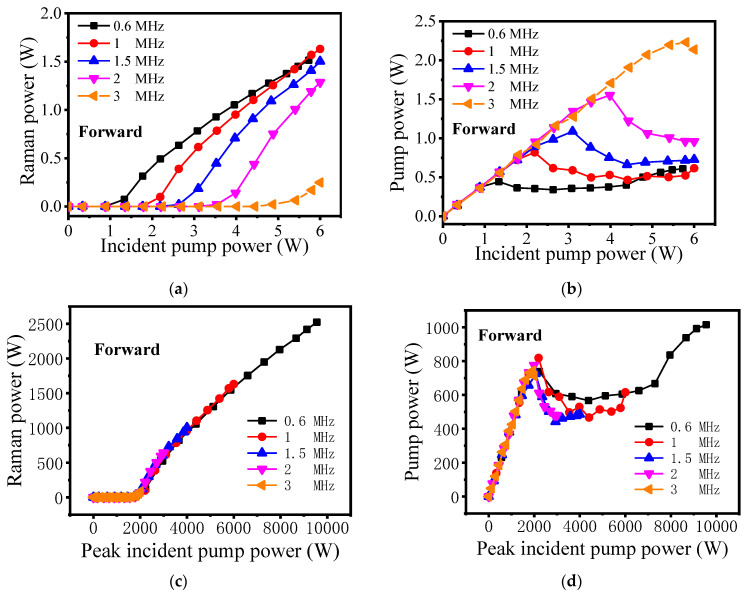
The characteristics of the forward output power with the incident pump power: (**a**) Raman power. (**b**) Pump power. The characteristics of the forward peak output power with the peak incident pump power: (**c**) Raman power. (**d**) Pump power.

The output laser power characteristics are measured as shown in Figure 5. Thanks to the good power stability of the pump source, the output Raman power is relatively stable, which facilitates the experimental measurement. By changing the repetition frequency of the pump source, the maximum forward Raman power of 1.63 W is obtained when the incident pump power is 6 W and repetition frequency is 1 MHz, as illustrated in Figure 5a. The corresponding optical-to-optical conversion efficiency inside the gas cavity is 58%. However, the total conversion efficiency is only 27%, mainly due to the fusion splicing losses of splice1 and splice2. When the repetition frequency is higher than 1 MHz, the pulse has lower peak power, which causes the decrease in energy beyond the Raman threshold, and the Raman power also decreases. When the repetition frequency is 0.6 MHz, there are two possible reasons why the maximum Raman power decreases. One is that part of the first-order Raman power has been converted to second-order Raman power, which slows down its growth. However, the second-order Raman laser has much higher transmission loss, and it is completely lost in transmission. The other possible reason is that the proportion of continuous wave of the pump source increases gradually, which causes a decrease in the pump power that can be converted to Raman power. The results in Figure 5b also confirm this possibility. When the repetition frequency is 0.6 MHz, the forward pump power shown in Figure 5b decreases at first when exceeding the Raman threshold power, then increases gradually. This is mainly because the proportion of continuous wave of the pump source increases. For other forward pump powers with different repetition frequencies, they increase at first and decline later due to the Raman conversion. Figure 5c,d present the characteristics of the forward peak output power with the peak incident pump power, it can be seen that all the curves show the same trends and the Raman threshold peak power is a constant. This is not unexpected, as the Raman threshold of the peak power is independent of pulse repetition frequency, and it is mainly determined by the gas pressure of hydrogen molecules in the cavity and the characteristic of the HC-PCF [33].

**Figure 6:**
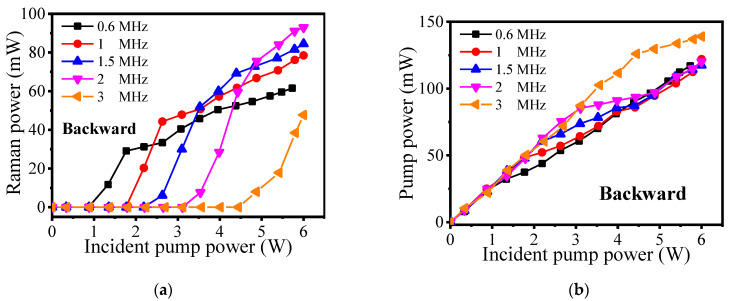
The characteristics of the backward output power with the incident pump power. (**a**) Raman power. (**b**) Pump power.

Figure 6a,b show the backward power characteristics of the Raman laser and pump power, respectively. It can be seen that both of the backward powers are far lower than the forward powers. For the backward Raman power in Figure 6a, the trend shows that with the increasing of the repetition frequency, the Raman power increases slowly. To further explore this, we calculated the ratio of backward Raman power to forward Raman power when the incident pump power is 6 W. The values are 4.07%, 4.82%, 5.63%, 7.2%, and 19.23%, with a repetition frequency of 0.6 MHz, 1 MHz, 1.5 MHz, 2 MHz, and 3 MHz, respectively. It means that high repetition frequency can be conducive to the amplifying of the backward Raman power. In Figure 6b, the backward pump powers keep increasing and tend to converge. This is because the backward pump power is from the Fresnel reflection of the splice1 and splice2 at first. With the increase in incident pump power, the pump power inside the HC-PCF is completely converted to Raman power, so the backward pump power is reflected by the splice1 at the high pump power level. By calculation, the backward pump power accounts for ~2.6% of the incident pump power, meaning the Fresnel reflectivity of splice1 is about 2.6%. According to the Fresnel formula R = [(1 − n)/(1 + n)]^2^, where R is the reflectivity and n is the refractive index, the theoretical value is supposed to be ~3.5%. This difference is mainly caused by the uneven surface of the splice1, which is not perfect specular reflection.

## 4. Conclusions

We have demonstrated the first all-fiber tunable pulsed 1.7 μm FGRL. When pumped by a homemade pulsed EDFA at 1.5 μm, the first-order Raman laser at 1.7 μm is obtained based on the rotational SRS of hydrogen molecules. By adjusting the repetition frequency of the pump pulse with a width of 10 ns, the maximum output power of 1.63 W is obtained. The corresponding optical-to-optical conversion efficiency inside the gas cavity is 58%, however, the total conversion efficiency is only 27%, mainly due to the relatively high splicing losses. By further reducing the splice loss [34], the output power and the conversion efficiency could be greatly improved. This work provides an elegant way to create all-fiber tunable 1.7 μm fiber lasers, and is also very significant for the development of FGRLs. 

## Data Availability

The data presented in this study are available on request from the corresponding author. The data are not publicly available due to privacy.

## Sample Availability

Samples of the compounds are not available from the authors.
